# The structure of immature tick-borne encephalitis virus supports the collapse model of flavivirus maturation

**DOI:** 10.1126/sciadv.adl1888

**Published:** 2024-07-03

**Authors:** Maria Anastasina, Tibor Füzik, Aušra Domanska, Lauri Ilmari Aurelius Pulkkinen, Lenka Šmerdová, Petra Pokorná Formanová, Petra Straková, Jiří Nováček, Daniel Růžek, Pavel Plevka, Sarah Jane Butcher

**Affiliations:** ^1^ Faculty of Biological and Environmental Sciences, Molecular and Integrative Bioscience Research Programme, University of Helsinki, Helsinki, Finland.; ^2^ Helsinki Institute of Life Sciences-Institute of Biotechnology, University of Helsinki, Helsinki, Finland.; ^3^ Central European Institute of Technology, Masaryk University, Brno, Czech Republic.; ^4^ Laboratory of Emerging Viral Infections, Veterinary Research Institute, Brno, Czech Republic.; ^5^ Department of Experimental Biology, Faculty of Science, Masaryk University, Brno, Czech Republic.; ^6^ Institute of Parasitology, Biology Centre of the Czech Academy of Sciences, Ceske Budejovice, Czech Republic.

## Abstract

We present structures of three immature tick-borne encephalitis virus (TBEV) isolates. Our atomic models of the major viral components, the E and prM proteins, indicate that the pr domains of prM have a critical role in holding the heterohexameric prM3E3 spikes in a metastable conformation. Destabilization of the prM furin-sensitive loop at acidic pH facilitates its processing. The prM topology and domain assignment in TBEV is similar to the mosquito-borne Binjari virus, but is in contrast to other immature flavivirus models. These results support that prM cleavage, the collapse of E protein ectodomains onto the virion surface, the large movement of the membrane domains of both E and M, and the release of the pr fragment from the particle render the virus mature and infectious. Our work favors the collapse model of flavivirus maturation warranting further studies of immature flaviviruses to determine the sequence of events and mechanistic details driving flavivirus maturation.

## Teaser

Molecular details of immature tick-borne encephalitis virus advance our understanding of how flaviviruses become infectious.

## INTRODUCTION

Tick-borne encephalitis virus (TBEV; *Orthoflavivirus encephalitidis*) belongs to the genus *Orthoflavivirus* and infects a range of ticks, birds, and mammals, including humans [reviewed in (*1*)], (*2*). The symptomatic infection in humans results in tick-borne encephalitis (TBE), a severe neurological disease that often results in long-term sequelae and can be fatal [reviewed in (*3*)]. The disease manifestations vary depending on the virus subtype, with the European subtype causing milder disease with a 0.5 to 2% mortality rate, the Siberian subtype often causing long-term or chronic infections with 1 to 3% fatality rate, and the Far Eastern subtype having the highest death rate of up to 35% (*4*). TBEV is endemic within Europe, Russia, and Northeast Asia (*5*). Despite the available vaccines, the number of infections has been steadily growing over the last few decades with about 10,000 to 15,000 annual reported cases [reviewed in (*6*)]. Specific therapies for TBE are not available.

TBEV is structurally similar to other flaviviruses (*7*, *8*) but has been less well studied than its mosquito-borne counterparts such as dengue (DENV), Zika (ZIKV), West Nile (WNV), Japanese encephalitis (JEV), and yellow fever (YFV) viruses [reviewed in (*9*, *10*)]. The virion consists of a single-stranded positive-sense RNA genome bound to multiple copies of the capsid (C) protein, surrounded by a host-derived lipid bilayer where 180 copies of membrane (M) and envelope (E) proteins are embedded, forming an icosahedrally symmetric shell. The building block of the shell in a mature virus is an E_2_M_2_ heterotetramer, with 90 such units lying parallel to the membrane in a smooth “herringbone” arrangement (*7*, *8*). TBEV enters the cells via receptor-mediated endocytosis where E plays a major role, mediating receptor binding and low pH-induced membrane fusion (*11*, *12*). Following uncoating, the viral genome is translated into a single multipass membrane polyprotein that is proteolytically processed to yield individual structural and nonstructural proteins. Viral RNA-dependent RNA-polymerase synthesizes negative-sense and subsequently positive-sense copies of the genome (*13*).

The newly synthesized genomes bind to multiple copies of C to form nucleocapsids (NC), which seem to acquire an envelope by budding into the lumen of the endoplasmic reticulum (*14*–*18*). The envelope of immature TBEV particle contains 180 copies of each M protein precursor (prM) and E forming heterodimers. The prM protein stabilizes E, preventing premature virus activation and its fusion at low pH with the membranes of the *trans*-Golgi network (TGN) (*19*, *20*). Immature particles of most flaviviruses are fusion-incompetent and noninfectious and must mature to acquire infectivity (*11*, *21*). Flavivirus maturation occurs at mildly acidic pH, when host protease furin cleaves the prM protein to a pr peptide that will later dissociate from the particle and the M protein that remains in the particle, resulting in infectious viruses (*22*, *23*).

Particle heterogeneity (*24*), flexibility (*25*), and symmetry imperfections (*26*) have limited the achievable resolution of immature flavivirus particle reconstructions. Detailed structural information is available only for the mosquito-borne Spondweni (SPOV) and mosquito-specific Binjari (BinJV*)* viruses, providing the first atomic details about the prME interactions (*27*, *28*).

Here, we used cryo–electron microscopy (cryo-EM), single-particle analysis, and localized reconstruction to determine the structure of immature TBEV particles (*29*–*33*). By analyzing two model TBEV strains—Hypr and Neudoerfl—and the Kuutsalo-14 isolate, we obtained comprehensive information about the architecture and molecular organization of immature TBEV and propose the major conformational changes that need to occur on the route to maturation.

## RESULTS AND DISCUSSION

To acquire a comprehensive understanding of the immature TBEV particle structure, we investigated three strains: Hypr, Neudoerfl, and Kuutsalo-14. We investigated the viruses at two different laboratories without coordinating the protocols for particle production, cryo-EM data collection, or image processing to obtain completely independent reconstructions. The immature particles were produced in infected cells treated with ammonium chloride to increase the pH of the exocytic pathway and thereby inhibit maturation (*21*, *22*, *34*). The purified samples contained primarily immature particles as indicated by the presence of a pronounced prM band on SDS–polyacrylamide gel electrophoresis (SDS-PAGE) (fig. S1). Before vitrification for cryo-EM, we inactivated the virus using formaldehyde or ultraviolet (UV) light that neutralized virus infectivity without affecting protein structure (*8*, *35*).

**Fig. 1.**
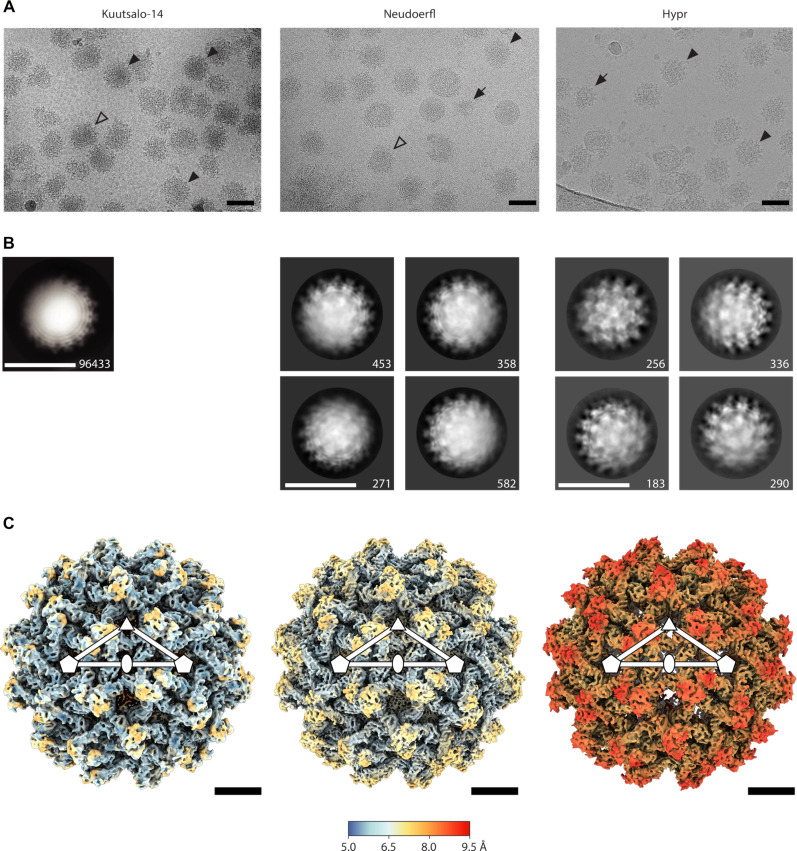
Cryo-EM and 3D reconstructions of immature TBEV particles. (**A**) Representative micrographs, where black arrowheads indicate spherical particles, white arrowheads indicate nonspherical particles, and black arrows indicate broken particles. Scale bars, 50 nm. (**B**) 2D class averages for Kuutsalo-14, Neudoerfl, and Hypr. The prevalent initial 2D class averages are shown below the respective micrographs with the number of particles per class indicated. Scale bars, 50 nm. (**C**) Isosurface representations of the icosahedral-symmetrized 3D reconstructions of immature Kuutsalo-14, Neudoerfl, and Hypr particles viewed down an icosahedral twofold axis of symmetry, colored by local resolution with the key indicated by the bar. Positions of selected symmetry axes are indicated by a white pentagon (fivefold), ellipse (twofold), and triangle (threefold) and an icosahedral asymmetric unit is outlined as a triangle. Scale bars, 10 nm.

The cryo-EM micrographs of all three strains revealed particles with a spiky appearance characteristic of immature flaviviruses (Fig. 1A). However, we observed considerable heterogeneity in particle structure, including broken particles, and particles with mixed spiky and smooth morphologies, which may represent partially mature particles. Reference-free two-dimensional (2D) class averages revealed protein densities projecting outward from the particle surface, causing the spiky morphology. However, in some of the 2D classes, one side of the particle was blurred, with poorly resolved protein and lipid bilayer densities. This blurring could be attributed to sample heterogeneity and/or symmetry imperfections within the particles (Fig. 1A). Imposition of icosahedral symmetry during the reconstruction enabled calculation of 7.1-Å-resolution maps of Kuutsalo-14 and Neudoerfl, and an 8.6-Å-resolution map of Hypr according to the 0.143 gold standard criterion of the Fourier shell correlation (GSFSC) cutoff (*36*) (Fig. 1B, figs. S2 to S4, and Table 1). The reconstructed particles contain 60 nonsymmetrical prM_3_E_3_ “spikes” (Fig. 1B).

**Table 1.** Cryo-EM data collection, refinement, and validation statistics. N/A, not applicable.

	Kuutsalo-14 full particle	Kuutsalo-14 prM_3_E_3_ spike	Neudoerfl full particle	Neudoerfl prM_3_E_3_ spike	Hypr full particle
Data collection and processing					
Magnification	81,000	81,000	105,000	105,000	75,000
Voltage (kV)	300	300	300	300	300
Electron exposure (e^−^/ Å^2^) total dose	43	43	40	40	69
Defocus range settings (μm)	−0.7 to −2.7 at 0.2 step	−0.7 to −2.7 at 0.2 step	−1.0 to −3.0 at 0.2 step	−1.0 to −3.0 at 0.2 step	−1.0 to −3.0 at 0.2 step
Pixel size (Å)	1.075	1.075	1.563 (binned 1.875×)	0.8336	1.62 (binned 1.5×)
Symmetry imposed	I2	C1	I1	C1	I1
Micrographs (*n*)	40,138	40,138	11,246	11,246	2262
Initial particle images (*n*)	271,870	4,312,440	156,614	1,639,578	29,978
Particles used in reconstruction (*n*)	70,189	1,172,996	36,236	552,993	18,160
Map resolution (Å)	7.1	3.9	7.0	4.0	8.6
FSC threshold	0.143	0.143	0.143	0.143	0.143
Map resolution range (Å)	999–7.1	999–3.9	999–7.2	999–4.0	999–8.6
Refinement					
Map sharpening *B* factor (Å^2^)	−658.6	−226.9/variable	−725	−167	−1178
Model composition					
Non-hydrogen atoms	N/A	14,700	N/A	13,407	N/A
Protein residues	N/A	1923	N/A	1908	N/A
E	N/A	1–496	N/A	1–492	N/A
prM	N/A	1–82, 85–98, 112–162	N/A	1–82, 86–95,111–160	N/A
Ligands	N/A	3 (NAG)	N/A	3 (NAG)	N/A
R.M.S.D.					
Bond lengths (Å)	N/A	0.66	N/A	0.002	N/A
Bond angles (°)	N/A	1.06	N/A	0.455	N/A
Validation					
MolProbity score	N/A	1.27	N/A	1.57	N/A
Clashscore	N/A	1.57	N/A	5.31	N/A
Poor rotamers (%)	N/A	0.06	N/A	0.00	N/A
Ramachandran plot					
Favored (%)	N/A	94.68	N/A	95.85	N/A
Allowed (%)	N/A	5.32	N/A	3.99	N/A
Disallowed (%)	N/A	0.00	N/A	0.16	N/A
PDB ID	N/A	8PPQ	N/A	8PUV	N/A
EMD	17809	17808	17946	17947	17945

**Fig. 2.**
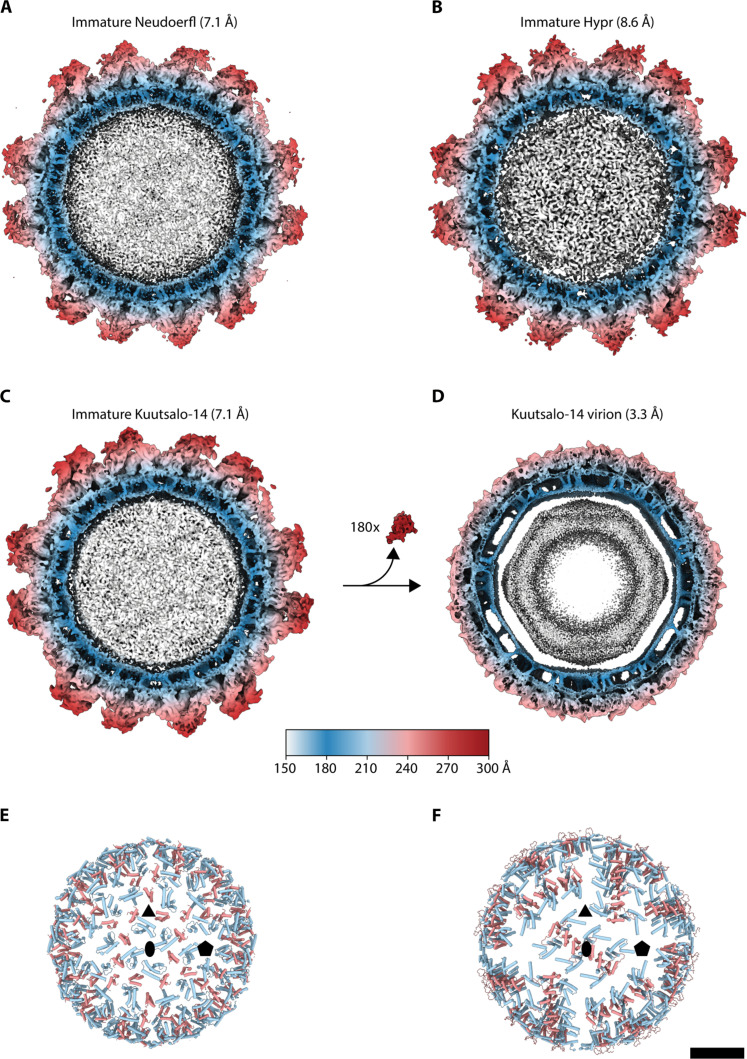
Membrane organization in immature versus mature TBEV particles. (**A** to **C**) Central sections (100 Å thick) of icosahedral reconstructions of immature particles compared to (**D**) the mature Kuutsalo-14 reconstruction (EMDB ID:14512) (*8*). Cleavage and dissociation of 180 copies of pr peptide upon maturation are indicated between (C) and (D). (**E**) Positions of transmembrane and peripheral membrane helices of E (light blue) and (pr)M (light red) in the front hemisphere of the immature Kuutsalo-14 and (**F**) in the mature Kuutsalo-14. Membrane-associated helices cluster into rafts in the mature particle compared to the more even distribution in the immature particle. Positions of selected symmetry axes in (E) and (F) are indicated by a black pentagon (fivefold), ellipse (twofold), and triangle (threefold). Scale bar, 10 nm.

**Fig. 3.**
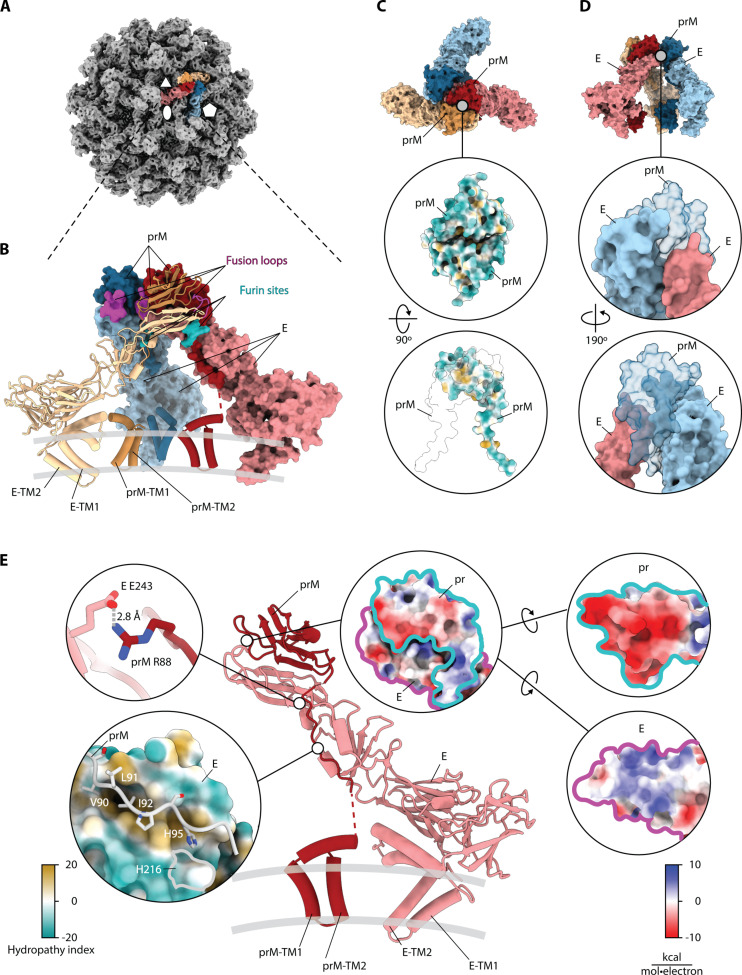
prM_3_E_3_ spike organization and protein-protein interactions. (**A**) Isosurface representation of the icosahedral reconstruction of immature Kuutsalo-14. Positions of symmetry axes are indicated using a white oval (twofold), triangle (threefold), and pentagon (fivefold). One prM_3_E_3_ spike is highlighted in color. (**B**) Atomic model of a Kuutsalo-14 prM_3_E_3_ spike refined against a 3.9-Å-resolution map. One prME heterodimer is shown in cartoon representation with TM helices of E and prM indicated. The remaining two prME heterodimers are shown as molecular surfaces. prM and E dimers are colored red, yellow, and blue with prM in darker shades. Fusion loops of E and prM furin cleavage sites are highlighted in magenta and turquoise, respectively. (**C**) A molecular surface representation of prM_3_E_3_ with a close-up view of the pr-pr interaction interface. Proteins are colored as in (B) in the top panel; both pr peptides are colored by hydrophilicity in the middle, and in the bottom panel, one pr peptide is colored according to hydrophilicity whereas the other prM is shown as a transparent molecular surface with black outline to indicate the interaction area. (**D**) A molecular surface representation of prM_3_E_3_ spike with a close-up view of E-prM-E interaction interfaces showing how one prM binds together the two E. The proteins are colored as in (B), but prM is semi-transparent. (**E**) A cartoon representation of one prME heterodimer with names of TM helices of pr and E indicated. Top left panel shows a close-up image showing a prME salt bridge. Bottom left panel shows a hydrophobic zipper stabilizing the furin site (E is colored by hydrophilicity, and prM is shown as string with relevant residues shown as sticks and labeled). The right side shows a close-up of a prME interaction interface colored by electrostatic potential (right side; individual proteins are outlined for clarity).

The structure of the NC is not resolved in the cryo-EM reconstructions of immature TBEV particles, indicating that it does not follow icosahedral symmetry. However, the transmembrane helices of E and prM traversing the lipid bilayer can be readily observed in cross sections of the icosahedrally symmetric reconstructions (Fig. 2, A to C). The ectodomains of E and prM form the spikes extending outward from the particle surface, contributing to the larger diameter of immature TBEV compared to the mature virion (~560 Å versus ~500 Å; Figs. 1B and 2, A to D). The mature virion exhibits a distinctly angular inner membrane leaflet, due to the clustering of the E and M protein transmembrane helices (Fig. 2D). In contrast, both membrane leaflets in the immature particle are round and the inner leaflet is more closely juxtaposed to the NC. This marked difference in the membrane shape is due to the different spatial organization of the transmembrane domains in mature and immature TBEV (Fig. 2, E and F). Furthermore, the transmembrane helices of E and M are slanted within the membrane in immature particles (Figs. 2C and 3B), contrasting to their orthogonal insertion in the virion membrane (Fig. 2D). Evidently, the topology of E differs between immature and mature particles, manifesting in unique arrangements of the ectodomain and membrane-associated regions, and greater projection of the E ectodomains outward from the particle surface in the immature TBEV. The membrane helix domains of both E and M can be unambiguously connected to their ectodomains in all three reconstructions. The connections in prM are similar to those found in BinJV (fig. S5), but not to those presumed in the immature Spondweni or DENV particles (*24*, *25*, *35*).

The interpretability of the icosahedral reconstructions of the whole immature TBEV particles was limited by the resolution (Figs. 2, A to C, and 3A; fig. S4; and Table 1). However, one can clearly identify the three prME heterodimers forming individual spikes at the particle surface (Figs. 1B , 2, A to C, and 3A). Suspecting the resolution was limited by particle heterogeneity (Fig. 1A), we used localized reconstruction of individual spikes and were able to improve the resolution for Kuutsalo-14 to 3.9 Å and the resolution of Neudoerfl to 4.0 Å at the 0.143 FSC cutoff (fig. S4), and to build atomic models for both strains. We observed a 1.0-Å root-mean-square deviation (RMSD) between Kuutsalo-14 and Neudoerfl atomic models for the Cα of prM and E ectodomains. As the prM amino acid sequences of the two strains are identical, and the E sequences differ only in residue 128 (Lys in Kuutsalo-14 and Arg in Neudoerfl), we explain the observed differences by the resolution limit of the maps used for refinement (fig. S6). The regions contributing to higher RMSD are in β sheets of E-DIII and loops. The RMSD of the Cα of the membrane-associated helices was 2.1 Å, reflecting the lower resolution of these regions. Localized reconstruction of Hypr did not result in an improved resolution compared to the icosahedral reconstruction and was not pursued further.

Each spike decorating the surface of immature TBEV particle is formed by three prME heterodimers and is asymmetric with two prME opposing each other (Fig. 3, B and C, red and yellow dimers), and a third dimer joining from the side (Fig. 3, B and C, blue dimer; movie S1). The peripheral (PM) and transmembrane (TM) helices of prM and E embed the proteins in the lipid bilayer. The E ectodomains extend outward from the particle surface joining at the top like a tripod, with the pr domains of prM positioned at the top (Fig. 3, A and B). The ectodomains of pr and E in the immature TBEV display the characteristic fold of flavivirus proteins, where pr comprises a globular beta-sandwich and E consists of three distinguishable domains (DI, DII, and DIII) (Fig. 3B and fig. S7). Our models reveal a discrepancy in the E ectodomain conformation compared to the recently published structure of prME crystallized at pH 4.6 (*20*) (fig. S7). In the latter, E-DII adopts a different position relative to DI resulting in a 19.5° difference with a total Cα RMSD of 8.6 Å between the models (*20*). The difference may arise due to the different oligomeric states of purified E ectodomains compared to the native E proteins within the particle, or due to the different probed pH. Furthermore, both TBEV (*20*) and DENV (*37*) prME crystal structures dimerized along the E-E interface. They include only the globular pr peptide complexed with E ectodomains, lacking the prM linker, the furin cleavage site, and both E and prM membrane-associated domains. This structure is distinct from the arrangement of prM_3_E_3_ in immature flaviviruses at neutral pH. These differences underline the importance of structural data obtained within the whole virus particle context, both at neutral and acidic pH.

The E-DII harboring the fusion loops join at the top of the prM_3_E_3_ spike and are capped by pr peptides, each of which binds to its respective E through an interface with a cumulative buried area of ~4700 Å^2^. The negatively charged prM surfaces cover the positively charged E-DII tips, obscuring the fusion loops (Fig. 3, B and E, and movie S1). The heterohexamer structure of the spike is stabilized by pr-pr interactions, as direct E-E contacts in the spike are limited to just one small interaction surface of ~160 Å^2^ (movie S1). The two opposing prME dimers interact via a symmetrical pr-pr interface with ~460 Å^2^ buried surface area (Fig. 3C), involving hydrophobic residues Ala^19^, Ala^20^, Val^31^, Leu^33^, Val^59^, Val^61^, and Phe^64^ of both prM proteins. The third prME dimer joins from a side via a ~160-Å^2^ interface between its E protein and E from another dimer and a ~320-Å^2^ interface between its pr and E of another dimer (Fig. 3D). Hydrophobic residues are enriched in both interfaces. Thus, the prM_3_E_3_ arrangement of the immature TBEV particle is mostly maintained by the interactions between pr domains within the prM_3_E_3_ spike. Analysis of E and M protein sequences of 182 TBEV isolates belonging to all three virus subtypes indicates that the interacting residues are highly conserved (*8*).

The globular pr domain of prM is connected to the membrane-associated helices by a flexible linker (fig. S5). We were able to build the linker along the E protein up to Gly^98^, including the conserved 85-Arg Thr Arg Arg-88 furin recognition site (*23*). The cleavage of prM at Arg^88^ splits it into pr peptide (residues 1 to 88) and M protein (residues 89 to 162) and is a crucial step of TBEV maturation (*23*). The residues forming the furin cleavage site are held in place by a polar interaction between Arg^88^ of pr and Glu^243^ of E, and is zipped downstream by the hydrophobic residues Val^90^, Leu^91^, and Ile^92^ of prM fitted into a hydrophobic pocket on E (Fig. 3E). This hydrophobic zipper is maintained in mature TBEV, where Val^2^, Leu^3^, and Ile^4^ of M (corresponding to Val^90^, Leu^91^, and Ile^92^ of prM) are docked into a hydrophobic pocket of E underneath E-DII (fig. S8), suggesting that the interaction is stable throughout the conformational rearrangement of prME and prM processing. An earlier hypothesis suggested that prM is cleaved after the E molecules fold into the herringbone pattern on the particle surface (*38*). The location of the M protein N terminus beneath the E-DII in the virion suggests that prM cleavage precedes herringbone structure formation, as the furin cleavage site would otherwise be inaccessible [fig. S8 and (*8*)]. Compared to the low pH structure of a pr fragment complexed with preassembled E ectodomain dimers (*20*), even there, the furin cleavage site would be inaccessible from the surface of the virus.

**Fig. 4.**
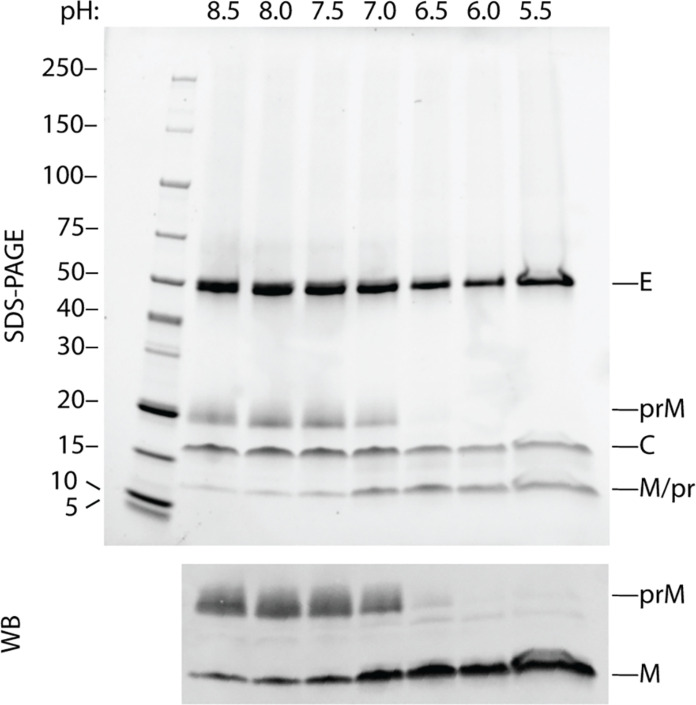
Susceptibility of immature TBEV to furin at decreasing pH. An SDS-PAGE of purified immature TBEV shows the three major protein components, E, prM, and C. As the particles are exposed to decreasing pH in the presence of furin for 16 hours at 30°C, prM is cleaved into pr peptide and M with increasing efficiency (top), which is verified by immunostaining with an antibody against the C terminus of M, as pr and M have similar molecular weights (bottom).

Within the immature particle context, the furin cleavage sites are located “inside” the spike (Fig. 3B and movie S1) and are inaccessible to the globular 88-kDa furin (*39*) without a conformational rearrangement of the spike. In vitro, the susceptibility of prM in immature TBEV to furin cleavage increases at pH 7 and below, indicated by a reduction of the prM, and the emergence of the M protein bands on SDS-PAGE, verified by Western blot (Fig. 4). The proteolysis is likely facilitated by the conformational changes of the spike that increase the cleavage site accessibility, as furin is known to be efficient throughout all our tested pH values (*22*, *40*) (Fig. 4). This pH-triggered conformational change of the spike agrees with earlier observations of pH-induced changes in the immature particle antigenicity (*19*). There are 15 conserved His in TBEV prME. A strictly conserved His^95^ from the prM linker interacts with the E hydrophobic pocket (Fig. 3E). This His is located within 4.5 Å of the conserved His^216^ in E, and we speculate that when protonated, these residues cooperate to trigger exposure of the loop containing the furin cleavage site to allow the conformational changes leading to proteolysis. Protonation of a single histidine in the prM linker has been proposed as a maturation trigger for DENV (His^98^) and SPOV (His^101^). In BinJV, the corresponding residue (His^100^) has been proposed to be an anchor point of M-E interaction during maturation [fig. S9 and (*27*, *28*, *41*)].

The prM linker (residues 99 to 111) is flexible and is not resolved in our prM_3_E_3_ maps (Fig. 3B and movie S1). However, densities connecting the globular pr domain to membrane helices of prM are evident in the icosahedral reconstructions of all the three immature TBEV particles (fig. S5), allowing us to assign the positions of prM membrane domains beneath the spike tripod (Fig. 3B). None of the spikes in the icosahedral reconstructions sit directly on the imposed symmetry axes, and thus, all the linkers can be treated as independent observations.

**Fig. 5.**
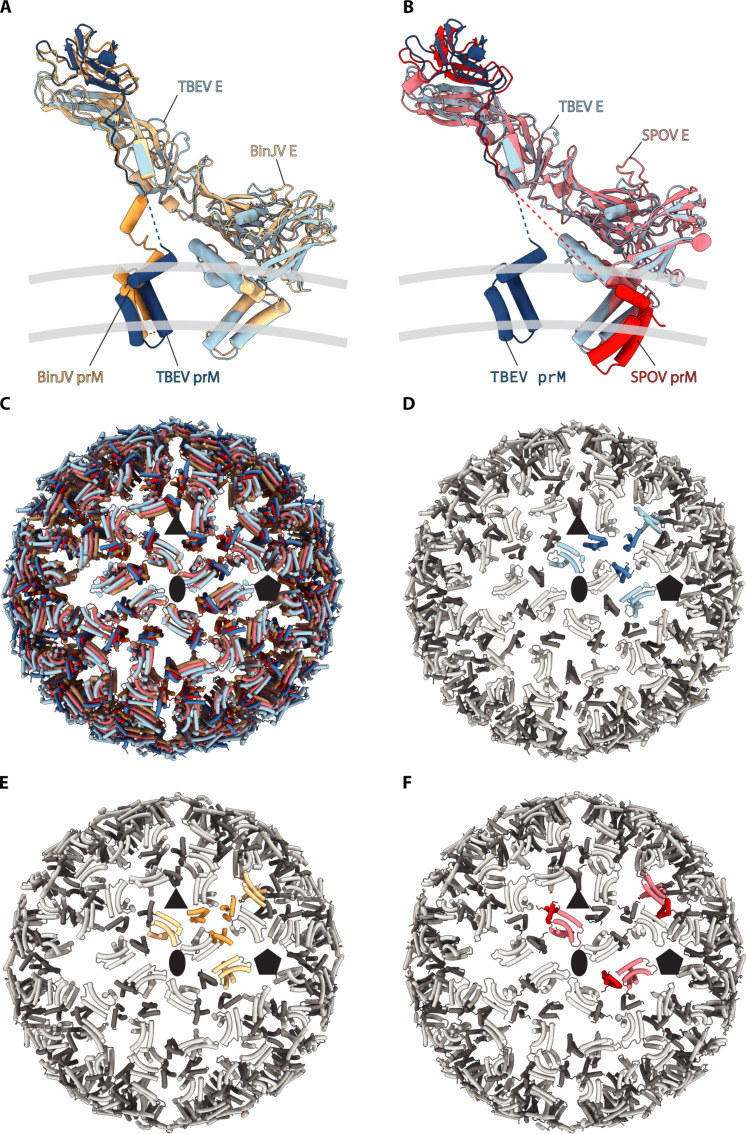
Comparison of membrane-associated domains of immature TBEV, BinJV, and SPOV particles. Cartoon representations of Kuutsalo-14 prME (blue) overlaid with (**A**) BinJV (yellow; PDB ID: 7l30) or (**B**) SPOV (red; PDB ID: 6zqj) show that prM membrane-associated domains are localized underneath the spike in TBEV and BinJV but are clustered with E membrane-associated domain in SPOV. (**C**) Only the transmembrane and peripheral membrane helices of E and prM are shown for the front hemispheres of immature TBEV (blue), BinJV (yellow), and SPOV (red) particles emphasizing similar distribution of membrane-associated domains in all three viruses. (**D** to **F**) Only the transmembrane and peripheral membrane helices of E (tints) and prM (shades) are shown for the front hemispheres of immature TBEV (D), BinJV (E) and SPOV (F). Membrane-associated domains of E and prM of one asymmetric unit per particle are highlighted in color, emphasizing similar domain assignment in TBEV [(D), blue] and BinJV [(E), yellow], but different in SPOV [(F), red]. Positions of selected symmetry axes are indicated by a black pentagon (fivefold), ellipse (twofold), and triangle (threefold).

A surprisingly dominant hypothesis for flaviviruses is that the prM linker downstream of the furin cleavage site extends along E, leading to an unproven topology for the membrane domains of prM and E (*37*). This unproven topology has been assumed in several models of immature flaviviruses, including DENV (*42*), ZIKV (*25*, *43*), and a recent high-resolution model of SPOV (*27*) (Fig. 5B). The only high resolution study to date with unambiguous topology is that of BinJV (*28*), which positions prM membrane-associated domains beneath the spike disproving the earlier hypothesis (Fig. 5A). We compared the overall TM domain distribution across the virus particle in TBEV, SPOV, and BinJV observing similar domain distribution for all the three viruses (Fig. 5C). As proposed earlier, we expect that the membrane domain topology is similar in all of the immature flaviviruses with the evidence coming from the visible prM linker density for both TBEV and BinJV (Fig. 5, D to F). This is in contrast to the topologies and domain assignments previously reported for other flaviviruses (checked within the wwPDB), including DENV and SPOV (*27*, *28*, *42*, *43*). Further studies are needed to determine the prM linker position, assign the TM domain positions, and interpret the movements required for flavivirus maturation.

The mechanism of particle maturation is a central question in flavivirus biology, which may be addressed through the interpretation of immature and mature particle structures and aided by biochemical data (*7*, *8*, *19*, *20*, *22*, *23*, *27*, *28*, *44*). The events driving maturation require destabilization of prM-prM interactions at the tip of the spike, exposure of the furin site in prM and its cleavage, and collapse of E ectodomains onto the membrane accompanied by the large movement of the E and M TM domains. Our structural data support the collapse concept of flavivirus maturation, where E ectodomains are prone to reposition parallel to the particle membrane as soon as the pr-pr interactions at the tip of the spike are disrupted (*28*). In TBEV, this conformational change is irreversible (*22*). The immature particle heterogeneity (Fig. 2A), the prM_3_E_3_ spikes’ flexibility, and the pH-sensing histidines in the proximity of the furin cleavage sites (Fig. 3E) facilitate local conformational changes at acidic pH to enable prM cleavage. The collapse of the spikes and the following major change of the particle morphology to the herringbone arrangement may therefore occur after pr dissociation. As the ammonium chloride treatment of the cells used to produce immature particles raises the exocytic pathway pH, based on this and the in vitro furin sensitivity assay (Fig. 4), the conformational changes enabling furin cleavage occur at pH 7 and below (*22*).

**Fig. 6.**
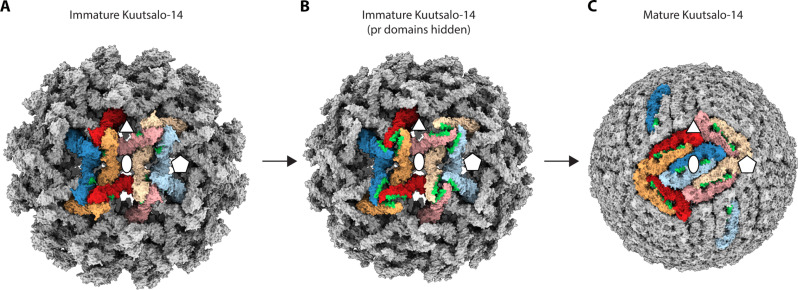
Redistribution of E ectodomains after TBEV maturation. (**A**) Surface representation of an immature Kuutsalo-14 particle is shown with two asymmetric units highlighted in color, and individual prME dimers are colored in tints and shades of red, yellow, and blue for clarity. (**B**) Same as in (A), but pr domains are hidden so that the E-E interacting surface is visible, highlighted in green. Note that dimerization surfaces on red and yellow E proteins are juxtaposed. (**C**) One of the possible redistributions of E on the virion surface after maturation collapse and dimerization of two E from one prM_3_E_3_ spike (red and yellow), whereas the third E (blue) will interact with one from the other prM_3_E_3_ (EMDB ID:14512) (*8*).

After spike dissociation, the E dimer formation is probably driven by the large dimerization surfaces of E proteins (*44*). Within one spike, two E proteins (red and yellow in Figs. 3, B and C, and 6, A and B) already have their dimerization surfaces facing each other. Although these E proteins have no interactions with each other in the immature spike, when they collapse onto the membrane, they would be readily positioned to stabilize each other by forming a dimer (red and yellow E proteins in Fig. 6C). The third E of that spike (blue in Figs. 3, B and C, and 6, A and B) will have to interact with the corresponding E of another spike; one such option is shown in Fig. 6 (A to C). The best molecular model of flavivirus maturation available so far is the BinJV one based on two end states (*28*). It describes that maturation requires the dissociation of pr-pr interfaces, an increase in furin site accessibility, movement in the TM region, and the prME collapse onto the membrane. An updated model could take into account the sequence of proteolytic cleavage and emphasize the possibility that maturation could start from a nucleation point and spread across the particle, minimizing protein clashes. The large observed heterogeneity of the immature TBEV particles (Fig. 1A) indicates a likely scenario where conformational change initiates at the most dynamic (disordered) region on the surface even at pH 7, allowing maturation to propagate across the surface as has been earlier proposed for DENV (*45*). In so doing, there could be an additional opening up of the prME that allows better proteolytic access to the furin cleavage site than either of the two endpoint models currently reveal. prM cleavage, the collapse of E protein ectodomains onto the virion surface concurrent with the large movement of the membrane domains of both E and M, and the release of the pr fragment from the particle render the virus mature and infectious. This knowledge contributes to our understanding of the flavivirus life cycle and may provide an explanation as to why the maturation of TBEV is irreversible (*22*). It can be aided with future studies on the pH-activated immature particle and timing of pr dissociation.

## MATERIALS AND METHODS

### Cells and viruses

Human neuroblastoma SK-N-SH cells (ATCC HTB-11) were maintained in Dulbecco’s modified Eagle’s medium (DMEM; Sigma-Aldrich) with glucose (1 mg/ml) supplemented with 10% fetal bovine serum (FBS, Gibco), penicillin (0.5 mg/ml), streptomycin (500 U/ml; penstrep; Lonza Bioscience), 2 mM glutaMAX (Gibco), and nonessential amino acids (1:100; NEAA; Gibco). Baby hamster kidney cells (BHK-21, ATCC CCL-10) were maintained in DMEM (Sigma-Aldrich) supplemented with 10% FBS (Sigma-Aldrich). All cells were maintained at +37°C at a 5% CO_2_ atmosphere.

TBEV strain Kuutsalo-14 (European subtype; GenBank MG589938.1) was a gift from O. Vapalahti, University of Helsinki. The virus stock was produced in SK-N-SH cells and titered using a plaque assay as described previously (*8*). TBEV strain Hypr (European subtype; GenBank U39292.1) was passaged five times in the brains of suckling mice and once in BHK-21 before its use in the present study. The virus was provided by the Collection of Arboviruses, Biology Centre of the Czech Academy of Sciences (https://arboviruscollection.bcco.cz). TBEV strain Neudoerfl (European subtype; GenBank U27495.1) was passaged several times in the brains of suckling mice, in UKF-NB-4 and BHK-21 cells before its use in the present study. The virus was provided by F. X. Heinz, Medical University of Vienna. The Neudoerfl and Hypr titers were estimated by plaque assay as described previously (*46*).

### Production and purification of immature TBEV

For production of Kuutsalo-14 immature particles, SK-N-SH cells were grown to 90% confluency, and the virus was added in the infection medium (DMEM, 2% FBS, glutaMAX, penstrep, NEAA, and 0.35 μM rapamycin) at a multiplicity of infection (MOI) of 10. At 22 hours post infection (h.p.i.), cells were washed with phosphate-buffered saline (PBS) and a fresh infection medium containing 20 mM NH_4_Cl was added. At 24 h.p.i., the procedure was repeated. The supernatant containing immature TBEV particles was collected at 48 h.p.i. and precleared by centrifugation at 4000*g* for 5 min. Immature TBEV was pelleted by centrifugation through a 30% sucrose cushion in HNE buffer (20 mM Hepes, pH 8.5, 150 mM NaCl, and 1 mM EDTA) at 131,000*g* at +4°C for 2 hours. The supernatant was discarded and the pellet was resuspended in HNE, treated with 25 U of benzonase (MerckMillipore) and immediately loaded onto linear glycerol-potassium tartrate gradients [30% glycerol–10% glycerol, 35% potassium tartrate (w/v)]. Following a 2-hour centrifugation at 126,500*g* at +4°C, particle-containing light-scattering bands were collected. The samples were concentrated and buffer exchanged to HNE using Amicon Ultra centrifugal filters (Merck) and irradiated with 25 mJ/cm^2^ of UV_245nm_ to inactivate infectivity. The protein concentration was determined using a Qubit Protein Kit (Thermo Fisher Scientific), and the protein content was analyzed using SDS-PAGE and immunoblotting as described previously (*8*).

For production of Hypr and Neudoerfl immature particles, BHK-21 cells were grown to 85% confluency and infected by TBEV strain Hypr or Neudoerfl at an MOI of 0.1 and 1, respectively. The cells were incubated in medium (DMEM, 5% FBS, and 25 mM Hepes, pH 7.4) for 18 hours (Hypr strain) or 26 hours (Neudoerfl strain). For the Hypr strain, a lower MOI and a lower infection time were chosen, because of the higher lytic effect of the viral strain on the BHK-21 cell line. The medium was replaced by medium containing NH_4_Cl (DMEM, 2% FBS, 25 mM Hepes, and 20 mM NH_4_Cl, pH 7.4), and the cells were incubated for 24 hours at +37°C, 5% CO_2_. The time of incubation was optimized to maintain infected cell viability while producing a sufficient amount of immature TBEV particles for cryo-EM analysis.

After incubation, the medium was clarified by centrifugation (5700*g* at +4°C for 20 min) and polyethylene glycol (PEG) 8000 dissolved in TNE buffer (20 mM tris, 120 mM NaCl, and 1 mM EDTA, pH 8.5) was added to the clarified supernatant. The final concentration of PEG was 8% (w/v). The particles were then fixed by the addition of 0.05% formaldehyde (v/v, final concentration) and precipitated overnight in an orbital shaker at 130 rpm, +4°C. The precipitated particles were pelleted at 15,000*g* for 60 min at +4°C. The pellet was resuspended in 10 ml of TNE buffer containing 8% PEG 8000 (w/v) and pelleted by centrifugation again at 15,000*g* for 60 min at +4°C. The pellet was resuspended in 3 ml of TNE buffer, RNase A was added (10 μg/ml, final concentration), and the mixture was incubated for 15 min at 15°C. The suspension was centrifuged at 15,000*g* for 10 min at +4°C, and the supernatant was loaded on 10 to 35% (w/v) potassium tartrate step gradient and centrifuged at 175,600*g* for 2 hours at +4°C. The light-scattering band was collected. The sample was buffer-exchanged into TNE buffer by serial dilution and concentration using Amicon Ultra centrifugal filters (Merck).

### In vitro maturation

Each 15-μl in vitro maturation reaction contained 5 μg of purified, UV-inactivated immature Kuutsalo-14 TBEV in HNE adjusted to the indicated pH using 1 M 2-(N-morpholino)-ethanesulfonic acid hydrate (pH 5.0). Reactions were supplied with 30 mM CaCl_2_ and 2 U human recombinant furin (Thermo Fisher Scientific, RP-062) and incubated at +30°C for 16 hours. After incubation, half of each reaction was mixed with 4× Laemmli sample buffer and proteins were resolved in 4 to 20% gradient SDS-PAGE. Protein bands were visualized using stain-free imaging using Bio-Rad GelDoc EZ, and specific prM and M bands were visualized using immunoblotting with an anti-M antibody as described previously (*8*).

### Cryo-EM sample preparation

The samples were vitrified in liquid ethane on glow-discharged 200 copper mesh R1.2/1.3 Quantifoil holey carbon-coated grids with 2-nm continuous carbon on top (Jena Bioscience) using a Leica EM GP plunger at 85% humidity with 1.5 s blotting time. Both TBEV strain Neudoerfl and Hypr samples were vitrified in liquid ethane on holey carbon-coated copper grids (Quantifoil 2/1, mesh 300, Quantifoil Micro Tools GmbH) using Vitrobot Mark IV (Thermo Fisher Scientific). Samples were stored under liquid nitrogen until use.

### Cryo-EM data collection

Kuutsalo-14 data were collected at the SciLifeLab CryoEM Infrastructure Unit (Solna, Sweden) using an FEI Titan Krios microscope (Thermo Fisher Scientific) operating at 300 kV equipped with a Gatan K3 detector in counting mode at a nominal magnification 81,000×, resulting in a sampling rate of 1.075 Å/pixel. Movies were recorded in counting mode at a total dose of 43 e^−^/Å^2^ distributed over 50 frames using a defocus range of −0.7 to −2.7 μm (step 0.2 μm). EPU software (Thermo Fisher Scientific) was used for data acquisition. A total of 69,964 movies were collected. TBEV strain Hypr was collected on a Titan Krios microscope (Thermo Fisher Scientific) operating at 300 kV aligned for parallel illumination in nanoprobe mode equipped with a Falcon3EC direct electron detector. The micrographs were collected in integration mode at a nominal magnification of 75,000×, resulting in a 1.08 Å pixel size on the detector. The defocus range applied during the acquisition was −3 to −1 μm, and the total dose during the 1-s acquisition was 69 e^−^/Å^2^. The dose-fractionated acquisitions were saved as 39 fraction movies. EPU software was used for the data acquisition. A total of 2262 movies were collected. TBEV strain Neudoerfl was collected on a Titan Krios microscope operating at 300 kV aligned for fringe-free imaging and equipped with a Gatan K3 direct electron detector behind an energy filter (BioQuantum K3, Ametek) with 10 eV slit inserted. The micrographs were collected in counting mode at a nominal magnification of 105,000×, resulting in a 0.8336-Å pixel size on the detector. The nominal defocus range applied during the acquisition was −3 to −1 μm, and the total dose during the 2-s acquisition was 40 e^−^/Å^2^. The dose-fractionated acquisitions were saved as 40 fraction movies. SerialEM software (*47*). was used for the data acquisition utilizing the beam-tilt compensation upon image shift. A total of 11,246 movies were collected. Data for both Hypr and Neudoerfl strains were collected at the Cryo–Electron Microscopy and Tomography Core Facility CEITEC MU (Brno, Czech Republic). See Table 1 for full data collection statistics.

### Image processing

Kuutsalo-14 image processing was performed at the Finnish Center for Scientific Computing (CSC) supercomputing cluster using Scipion 3.0 framework and Cryosparc (*30*, *33*). Movies were aligned using MotionCor2 implemented in Relion 3.1 (*48*, *49*), and movies with per-frame motion exceeding 5 pixels were rejected using Xmipp movie maxshift (*50*). Contrast transfer functions were calculated using gctf and CTFFind4, and micrographs with resolution discrepancies beyond 3.0 Å, as well as with low resolution (worse than 6 Å) and astigmatism (>500 Å), were rejected using CTF consensus (*50*–*52*). Particles were picked and extracted with a box size of 900 pixels using Xmipp3, and several rounds of 2D classification were carried out using Relion 3.1.2. The selected particles (96,433) were used for initial model generation using the stochastic gradient descent method as implemented in Relion 3.1.2 (*48*). Particles were subsequently 3D classified and refined in Relion 3.1 or in Cryosparc using a 650-Å spherical mask to a final resolution of 7.10 Å according to the FSC_0.143_ criterion. For localized reconstruction of prM_3_E_3_ spikes, sub-particles were defined and extracted using localized reconstruction package integrated into Scipion (*53*) and exported using Relion export particles function. The sub-particles were imported into Cryosparc v4.2.1 and were reconstructed using iterative ab initio model generation, 3D classification, and refinement using a soft segment mask that included a single spike with the membrane part. The final resolution of the reconstructed spike was 3.89 Å according to the FSC_0.143_ criterion. The local resolution of prM_3_E_3_ map was estimated using Cryosparc and the map was locally sharpened using Xmipp3 or minimally sharpened using Relion 3.1 postprocessing function. Both the locally and the minimally sharpened maps were used for model building.

The TBEV strain Hypr data were processed as follows. The collected dose-fractionated movies were aligned using MotionCor2 (*49*), and the sums of dose-weighted fractions were saved as individual micrographs. CTF estimation was performed using Gctf v1.06 (*51*). The initial set of particles was manually picked on nonbinned micrographs and the crYOLO neural network was trained on this subset. Automatic particle picking was performed on micrographs using crYOLO (*54*). For initial 2D classification, the particles were extracted and downsampled from a 768-pixel box size to 128-pixel box size (final pixel size, 6.48 Å). Several rounds of 2D classification were performed in Relion 4.0.0 (*55*). The selected 18,160 particles were reextracted and downsampled to a box size of 512 pixels (pixel size, 1.62 Å). The stochastic gradient descent method as implemented in Relion 3.1.2 (*48*) was used for initial model generation. Refinement using the initial model low pass filtered to 40 Å was done in Relion v3.1.2 with icosahedral symmetry applied, using only a spherical mask of diameter 650 Å. No further 3D classification or masked refinement improved the map resolution or quality. The final map was masked by a threshold mask and *B*-factor sharpened in the postprocessing procedure using Relion 3.1.2. The final resolution was estimated using the FSC_0.143_ criterion as 8.64 Å.

The TBEV strain Neudoerfl data were processed as follows. The collected dose-fractionated movies were aligned using MotionCor2, and the sum of dose-weighted fractions were saved as individual micrographs. CTF estimation was performed using Gctf v1.06. The initial set of particles was hand-picked on 10× binned micrographs, and the crYOLO neural network was trained on this subset. Automatic particle picking was performed on 10× binned micrographs using crYOLO, and the resulting coordinates were corrected by the binning factor to match the particle positions on the unbinned micrographs. For initial 2D classification, the particles were extracted and downsampled from a 960-pixel box size to 128-pixel box size (final pixel size, 6.25 Å). Several rounds of 2D classification were performed in Relion 4.0.0 to exclude false-positive particles picked by crYOLO. The resulting 36,236 particles were reextracted and downsampled to a box size of 512 pixels (pixel size, 1.56 Å). Refinement using a low pass–filtered (40 Å) initial model from a previous refinement of TBEV Hypr was done in Relion v 3.1.2 with icosahedral symmetry applied, using only a spherical mask of diameter 650 Å. No further 3D classification or masked refinement improved the map resolution or quality. The final map was masked by a threshold mask and *B*-factor sharpened in the Relion 3.1.2 postprocessing procedure. The final resolution was estimated using the FSC_0.143_ criterion as 7.15 Å. To improve the resolution of the spike-trimers, single spike-trimers were extracted in a 300-pixel box from the original micrographs by a modified version of localized reconstruction (*32*). A total of 1,639,578 sub-particles were extracted and subjected to initial 3D refinement using local searches around the already known orientations. A soft segment mask that included a single spike-trimer together with the membrane was applied in the refinement step. After refinement, 3 rounds of 3D classification were performed, dividing the particles into 40 classes. The orientational search was omitted during the classification and the orientations from the previous refinement step were used. The selected classes included 552,993 particles, which were subjected to 3D refinement. This was followed by anisotropic magnification estimation, third- and fourth-order aberration estimation, and defocus refinement per particle in Relion 3.1.2. Finally, the volumes were reconstructed using relion_reconstruct with applied Ewald sphere correction. The final map was masked and *B*-factor sharpened. The final resolution was estimated using the FSC_0.143_ criterion as 4.03 Å.

### Model building

The homology model of Kuutsalo-14 prM_3_E_3_ was generated using I-TASSER (*56*) with BinJV prM_3_E_3_ (*28*) [Protein Data Bank (PDB) ID: 7l30] as a template. Membrane-associated domains of prM (residues 112 to 161) were replaced with membrane-associated domains of M from mature TBEV (*8*) (PDB ID: 7z51). The resultant model was flexibly fitted into the prM_3_E_3_ maps using ISOLDE integrated into ChimeraX (*57*, *58*). The model was real-space refined using Phenix version 1.20 (*59*), after which clashes were fixed in ISOLDE with model-based distance and torsions constraints imposed. For TBEV strain Neudoerfl, an Alphafold v2.2 (*60*) generated model of the E-prM complex multimer was manually rigid body fitted into the postprocessed electrostatic potential map of the prM_3_E_3_ spike using UCSF Chimera (*61*). The ectodomain of the E-protein and the prM fitted well and manual adjustments were done in Coot (*62*), followed by real-space refinement in Phenix v1.20. The membrane part of the E-protein was modeled using BinJV prM_3_E_3_ (*28*) (PDB ID: 7l30) E-protein transmembrane domain as template, and the membrane part of the prM was modeled using TBEV M protein (*7*) (PDB ID: 5O6A) as a template. Because of the low resolution of the map in the membrane region, the side chains of the amino acid residues in this region were stripped, and secondary structure restraints were applied before Phenix real-space refinement. The final model containing the joined ectodomains and transmembrane domains was iteratively refined using Phenix real-space refinement and the refined model was manually inspected and corrected in Coot, until final convergence. The geometry of all models was continuously monitored using MolProbity (*63*).
