# Supplementary material for: The structure of immature tick-borne encephalitis virus supports the collapse model of flavivirus maturation
Source: Sci Adv. 2024 Jul 3;10(27):eadl1888. doi: 10.1126/sciadv.adl1888 (PMC11221509; doi:10.1126/sciadv.adl1888)
Supplement: Supplementary file 1 — Figs. S1 to S9 [file sciadv.adl1888_sm.pdf]

Science Advances
AAAS

Supplementary Materials for

The structure of immature tick-borne encephalitis virus supports the collapse model of flavivirus maturation

Maria Anastasina *et al.*

Corresponding author: Pavel Plevka, pavel.plevka@ceitec.muni.cz; Sarah Jane Butcher, sarah.butcher@helsinki.fi

Sci. Adv. **10**, eadl1888 (2024)
DOI: 10.1126/sciadv.adl1888

The PDF file includes:

Figs. S1 to S9

Other Supplementary Material for this manuscript includes the following:

Movie S1

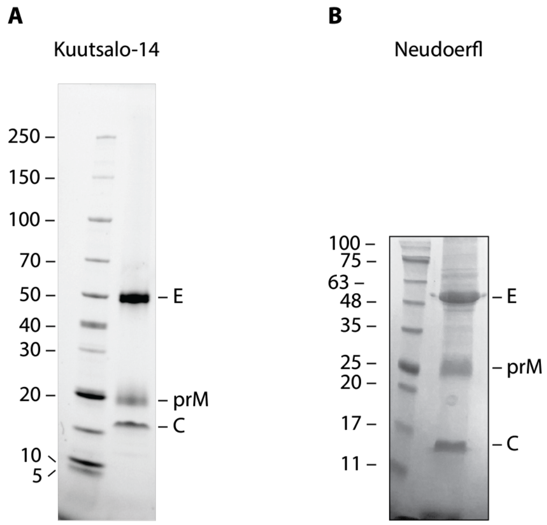

Figure S1. ***Purity of immature TBEV preparations.*** *SDS-PAGE of purified immature particles shows the 3 major protein components, E, prM, and C for Kuutsalo-14 (**A**) and Neudoerfl (**B**). Molecular weight markers are shown on the left.*

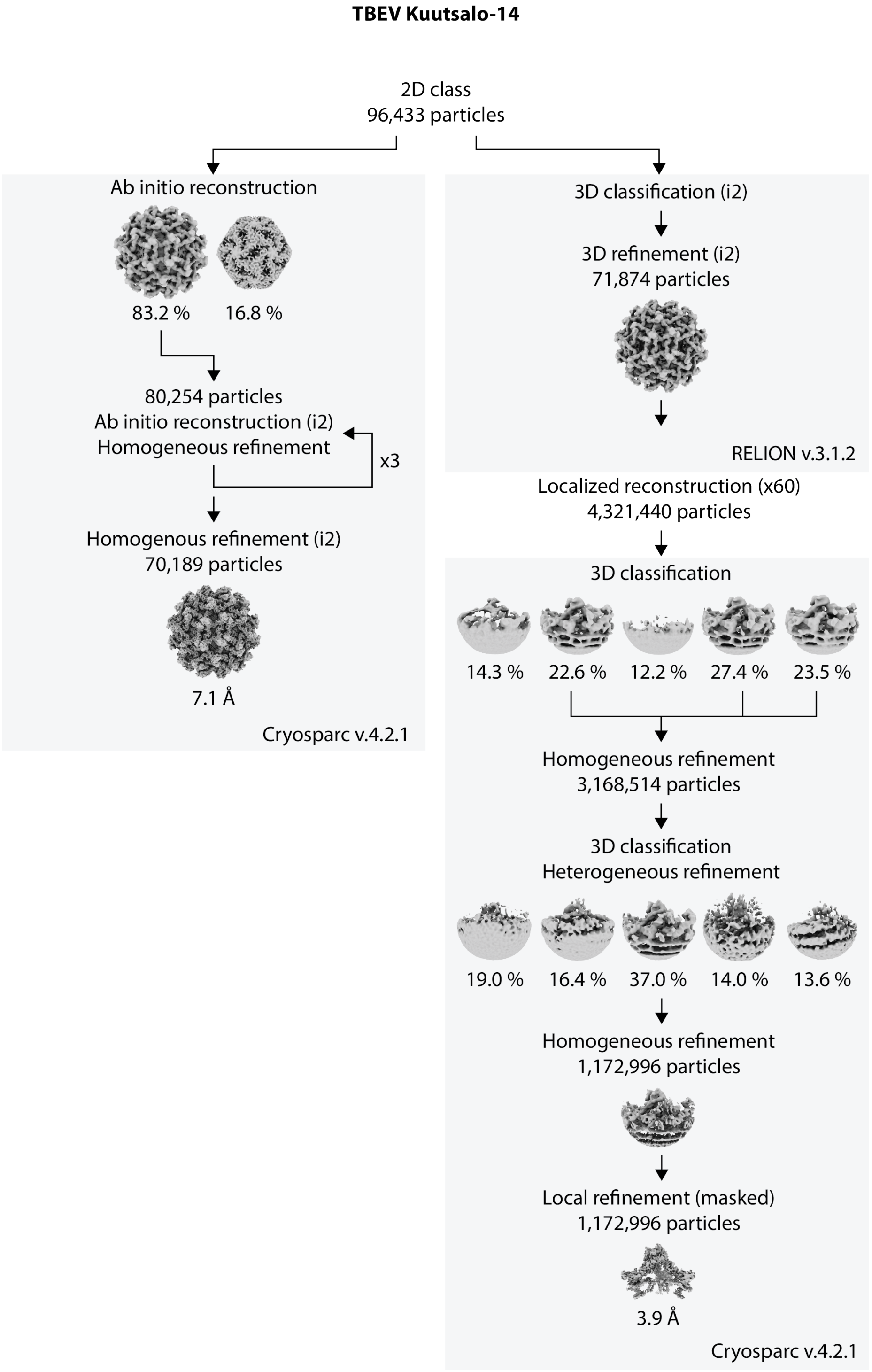

Figure S2. ***CryoEM data processing flow chart of immature Kuutsalo-14 TBEV.*** *Flowchart of processing steps of immature Kuutsalo-14 going from selected 2D class to icosahedral reconstruction of immature particles, and to localised asymmetric reconstruction and refinement of trimeric prM$_3$E$_3$ spike.*

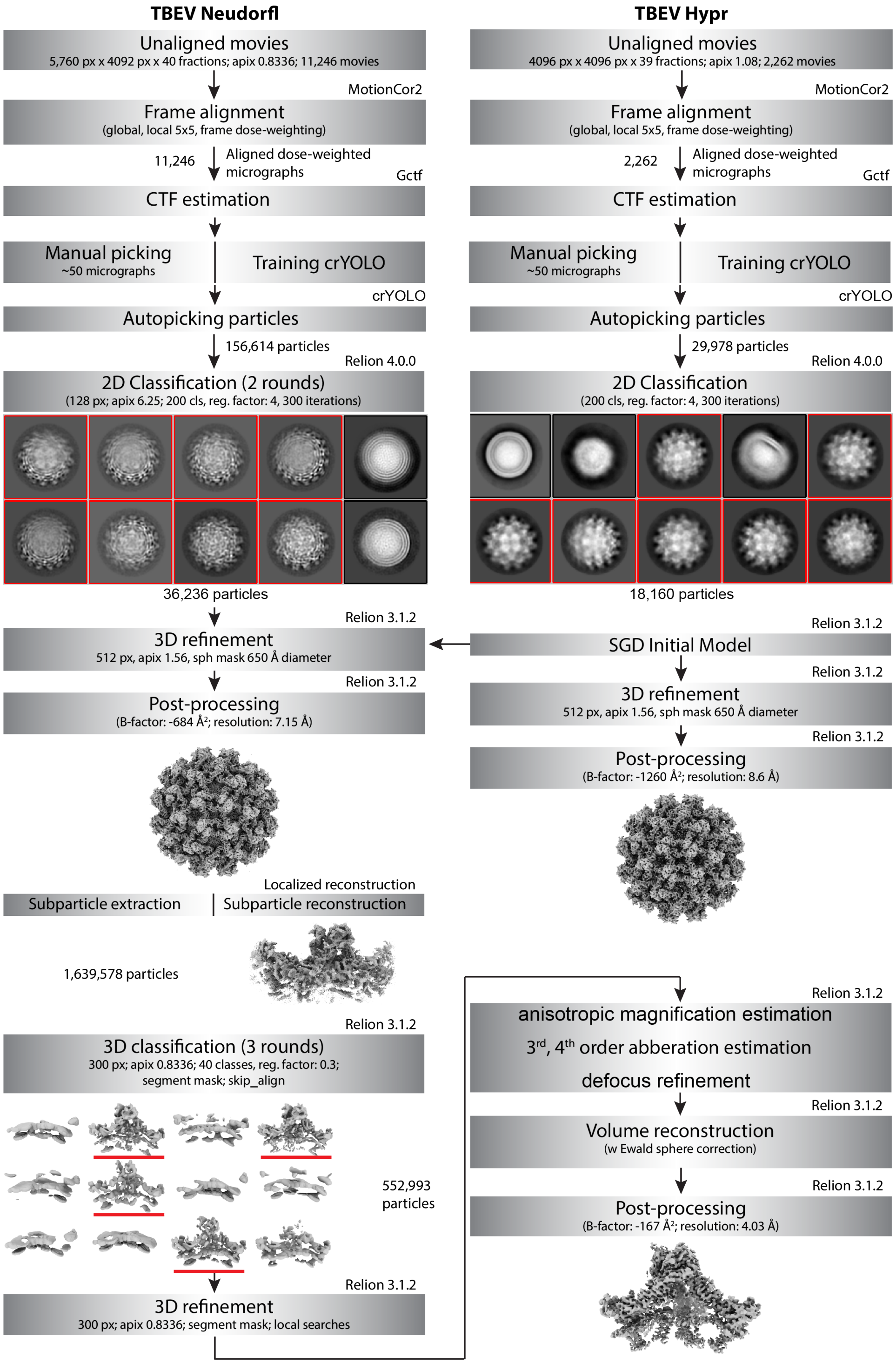

Figure S3. ***CryoEM data processing flow charts of immature Neudoerfl and Hypr TBEV.*** *Flowcharts of processing steps of immature Neudoerfl and Hypr TBEV starting from image pre-processing, particle picking, and classification, to the icosahedral reconstructions of immature particles for both strains and to localised asymmetric reconstruction and refinement of trimeric* prM_3E_3 *spike of the Neudoerfl strain.*

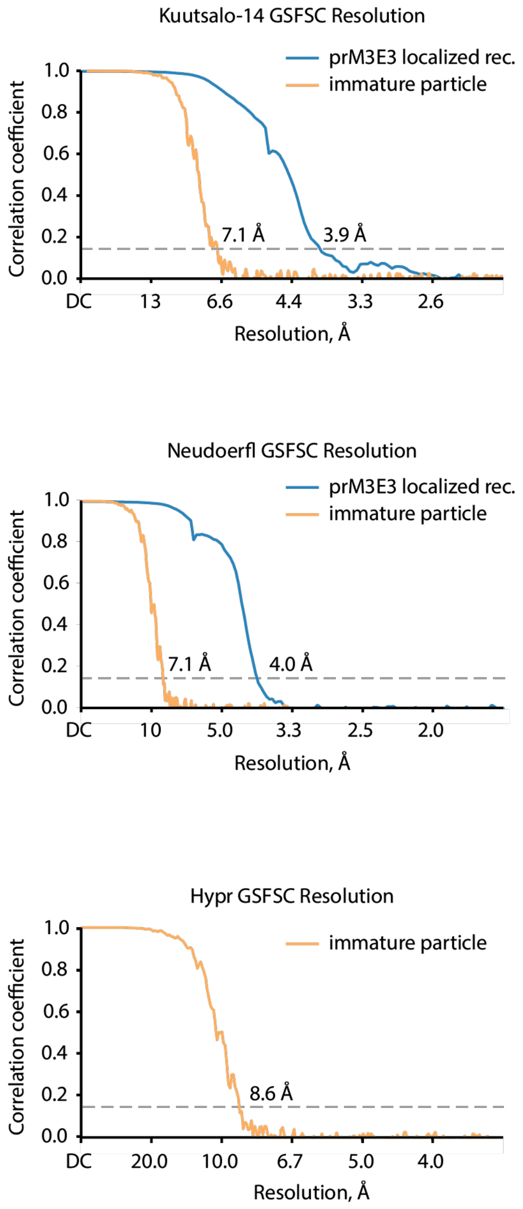

Figure S4. ***Fourier Shell Correlation curves of the half-reconstructions using gold-standard refinement in RELION and CryoSPARC.*** *The grey dashed line indicates a 0.143 FSC cutoff and approximate resolutions at this cutoff are indicated. The X-axis extends to the Nyquist resolution of the collected dataset. FSC curves are shown for masked half-maps.*

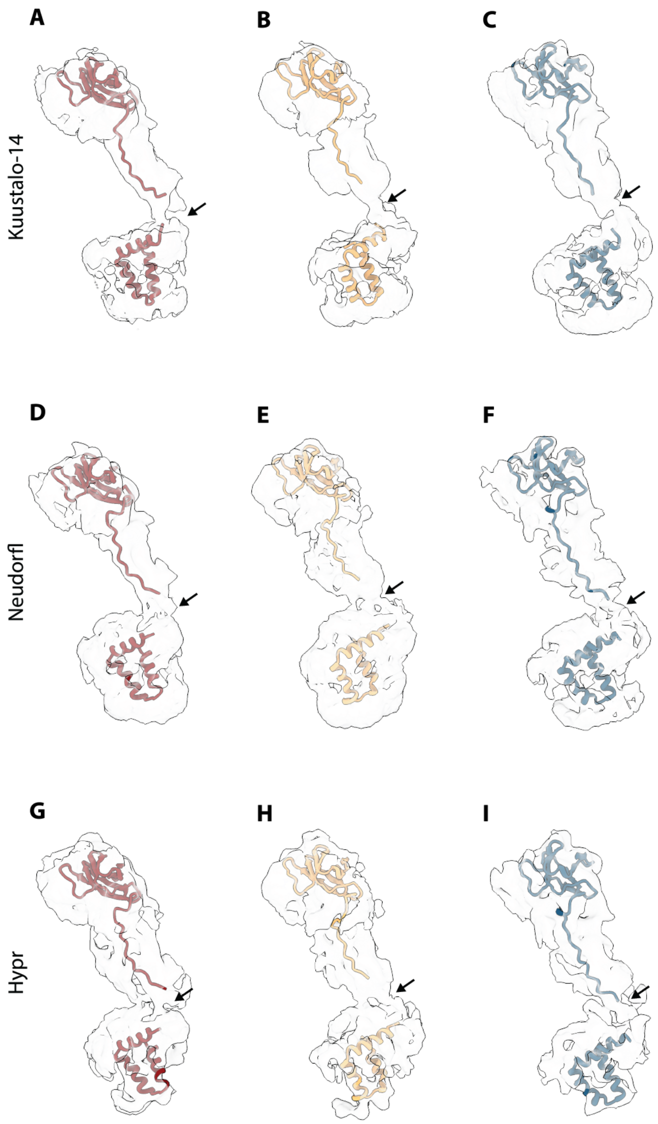

Figure S5. ***prM linker density observed in icosahedral reconstructions of immature TBEV.*** *Sections of whole particle density maps surrounding rigidly fitted atomic models of Kuutsalo-14 (**A-C)**, Neudoerfl (**D-I**). Individual prM chains are shown for each prM copy within an asymmetric unit with linker densities indicated with arrows.*

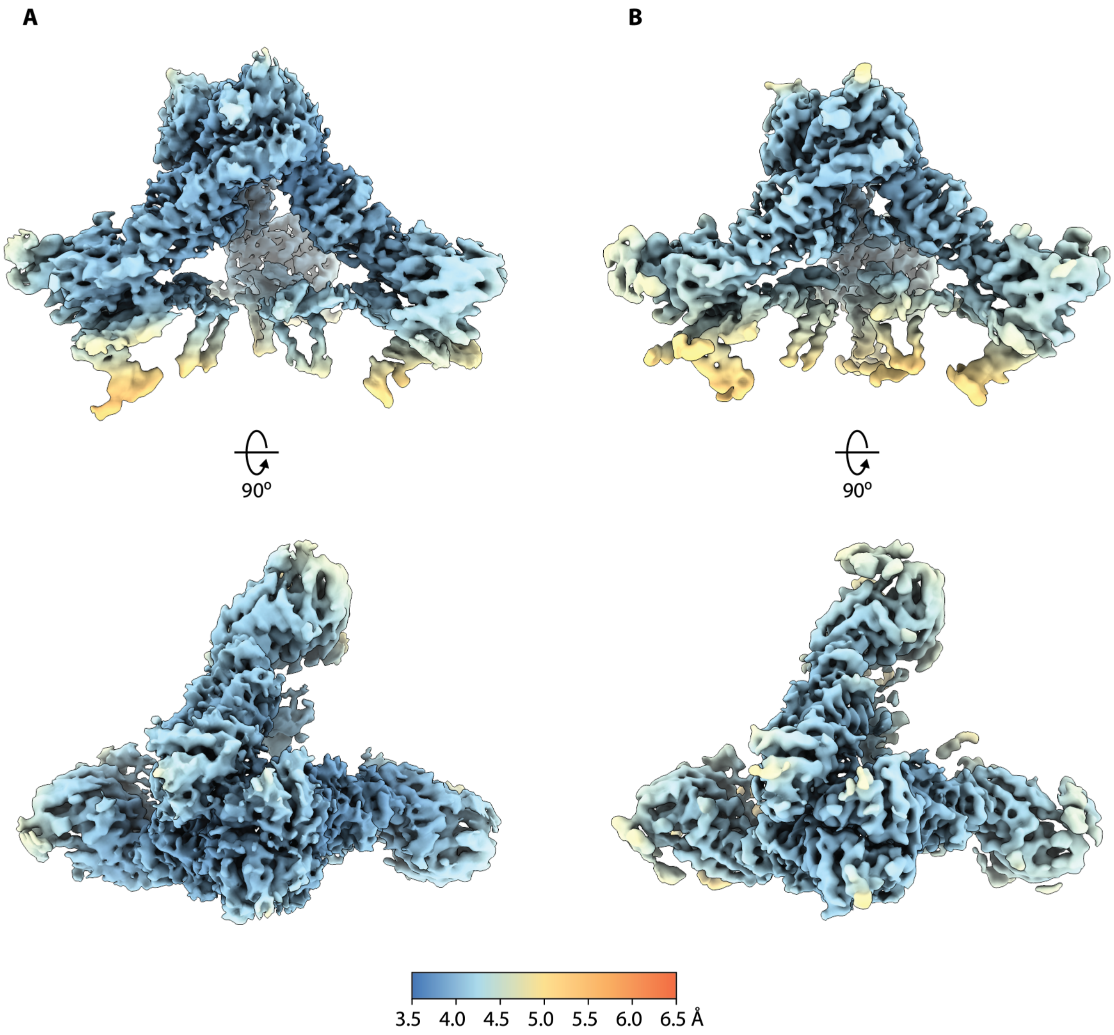

Figure S6. ***Isosurface representations of prM$_3$E$_3$ spikes*** *of the Kuutsalo-14 (A) and Neudoerfl (B). The spikes are coloured by local resolution with the key indicated by the bar.*

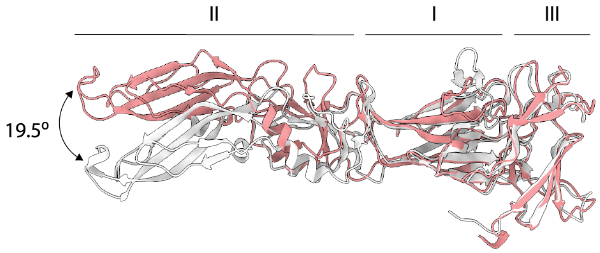

Figure S7. ***Comparison of sE of cryoEM and X-ray prME models.*** *An overlay of E protein ectodomain from Kuutsalo-14 prM$_3$E$_3$ (red) and from Neudoerfl (pr/sE)$_2$ crystallised at pH 4.6 (grey; PDB ID: 7QRE). Domains I, II, and III are indicated, and a 19.5° difference in the positions of domains II is indicated.*

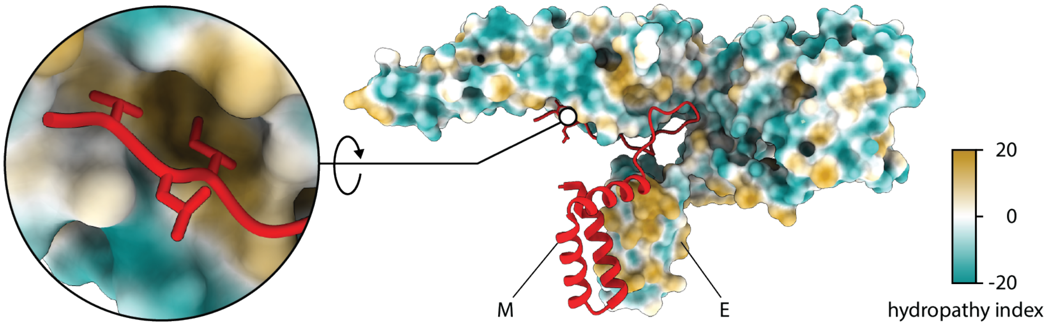

Figure S8. ***A hydrophobic zipper downstream of the furin cleavage site is maintained in the E-M dimer of the TBEV virion.*** *A surface representation of E coloured by hydrophilicity and a cartoon representation of M from TBEV virion (PDB ID: 7z51) show a stretch of hydrophobic residues Val2-Leu3-Ile4 on M docked into a hydrophobic pocket of E, proximal to the membrane. Residues Val2, Leu3, and Ile4 in M correspond to residues Val90, Leu91, and Ile92 of prM involved in stabilisation of the furin cleavage site in the immature TBEV.*

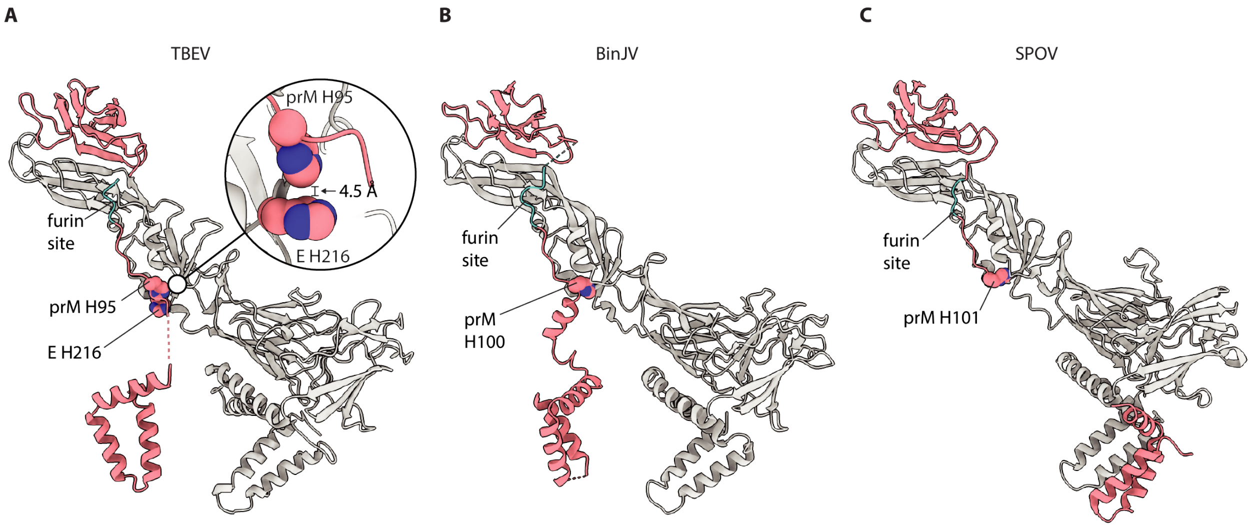

Figure S9. ***Histidines in the proximity of the furin cleavage site play a role in flavivirus maturation.*** *A cartoon representation of one prME dimer of* ***(A)*** *A cartoon representation of one TBEV prME dimer with the prM His95 and the E His216 histidines in the proximity of the furin site shown as spheres coloured by heteroatom. The close-up view in shows the proximity of prM His95 of and E His216 that located within 4.5 Å of each other* ***(B)*** *A cartoon representation of one prME dimer of BinJV (PDB ID: 7L30) with the His100 of prM shown as spheres, and* ***(C)*** *A cartoon representation of one prME dimer of SPOV (PDB ID: 6ZQI) with the H101 in prM shown as spheres. The position of the furin cleavage site is indicated. The E chains are shown in grey, and the prM chains are shown in pale red in all the panels.*
